# Experimental and Numerical Study on Dynamic Response of Foam-Nickel Sandwich Panels under Near-Field Blast Loading

**DOI:** 10.3390/ma16165640

**Published:** 2023-08-16

**Authors:** Pengzhao Xu, Ning Zhao, Yukun Chang, Shaokang Cui, Kunlin Shi, Bao Zhang

**Affiliations:** 1 School of Mechanical Engineering, Northwestern Polytechnical University, Xi’an 710072, China; 2 Xi’an Institute of Electromechanical Information Technology, Xi’an 710065, China; 3 School of Mechano-Electronic Engineering, Xidian University, Xi’an 710071, China; 4 Shaanxi Key Laboratory of Space Extreme Detection, Xi’an 710071, China

**Keywords:** explosive, blast load, protective, sandwich panels, foam-nickel, experimental testing, numerical simulation

## Abstract

The explosion products, such as shock waves, fragments and heat energy formed by explosion, act on the plate structure, which may cause structural damage, material failure and even phase transformation of material. In this paper, the damage mechanism and protective effect of near-field blast load on sandwich structure based on foam-nickel core material were studied. Firstly, the near-field explosion test was conducted to investigate the blast response of the foam-nickel sandwich structure subjected to blast shock from 8701 explosive at near-field position. The deformation characteristics and stress history of the sandwich structure on the acting location of blast load were carefully investigated via experimental methods. A finite element model of near-field explosion was established for effective numerical modelling of the dynamic behaviour of the sandwich structure using the explicit dynamics software ANSYS/LS-DYNA for more comprehensive investigations of the blast shock response of the sandwich structure. The finite element model is reasonable and validated by mesh independence verification and comparing the simulated response behaviour to that from the experimental results for the sandwich structure subjected to near-field blast load. On this basis, the damage mechanism and protection effect of the near-field explosion impact on foam-nickel cores with different density and porosity are simulated more systematically. The investigated results from the experiments and a series of numerical simulations show the large deformation effect due to the extensive energy absorption, which suggests that the sandwich structure based on foam-nickel core material may be expected to become a new choice of protective structure under near-field blast load.

## 1. Introduction

Porous metal sandwich structures have the advantages of light weight, simple construction, high specific strength and excellent energy absorption capacity, and are often used as energy-consuming components of ships, aircraft, automobiles and other structures, and are also used in the field of blast load protection [1,2,3,4,5]. As one of the typical porous metal sandwich structures, the metal foam sandwich board is composed of a metal panel, a backplane and a lightweight metal foam sandwich in the middle, and it has good impact resistance. When subjected to strong blast loads, it can effectively protect important structures placed behind the sandwich structure, such as people and equipment, which has attracted extensive attention from scholars at home and abroad [6,7,8,9,10,11].

Numerous studies have concentrated on the energy absorption properties and deformation characteristics of sandwich structures applied on multifarious loading environments [12,13,14,15,16,17]. Jing et al. [18] investigated experimentally the blast shock resistance of a thin shell of sandwich structures consisting of metal plates and gradient foam core layers under blast load, and relevant test results display the strong energy absorption capacity for the sandwich structures. Based on a three-point bending test, Avila [19] found that the layered sandwich beam has a better shock resistance when the top core layer has the highest density. Similarly, Gardner et al. [20] conducted a series of blast load tests for multiple gradient composite sandwich beams and explored the effect of the number of core layers on the blast dynamical response of the entire structure. Their results indicated that the increase in the number of core layers improves significantly the shock resistance of the entire structure. Recently, Ma et al. [21] compared the performances of blast dynamical response of aluminium honeycomb sandwich panels with fibre metal core laminate, and the results showed that the honeycomb sandwich panel has a more excellent blast resistance than the aluminium alloy skin sandwich panel. Jing et al. [2,22] carried out an optimization design for aluminium sandwich beams under drop-weight impact and blast load, respectively.

However, multiple investigations on the blast shock resistance of metal foam sandwich panels mainly focus on aluminium foam core from numerous literature studies; there are few reports on foam-nickel, which in fact may be a new energy absorption material owing to its porous structure. Therefore, it is of great significance to study the damage characteristics of foam-nickel sandwich plate under the action of explosion shock wave for the design of foam protective structure.

Compared to mid/far-field explosions, the blast waves generated by the near-field blast have long duration and short propagation distance, meaning that most of the detonation waves acted on the structure, which will cause distinctly different damage modes [23,24,25,26]. The dynamic response and failure mechanism of a sandwich protective structure under near-field blast load were experimentally and numerically investigated. In this paper, a sandwich panel based on foam-nickel core material was designed, and some of its main dynamical performances, including the deformation modes, blast response and energy absorption capacity, were investigated via experimental and finite element simulation approaches. Firstly, a near-field explosion test was conducted at an outdoor test place to investigate the blast response of the foam-nickel sandwich structure subjected to blast shock at near-field position. The deformation characteristics and stress history of the sandwich structure on the acting location of the blast load were carefully analysed based on the experimental results. Then, a finite element model of near-field explosion was established for series of the dynamic response analysis of the sandwich structure by using the explicit dynamics software ANSYS/LS-DYNA. The finite element model is reasonable and validated by mesh independence verification and comparison from test results. Finally, on this basis, the damage mechanism and protection effect of near-field explosion impact on foam-nickel core with different density and porosity were simulated more systematically, and the deformation and energy absorption mechanisms were further revealed. Therefore, this research may be expected to provide fundamental data and design guidelines for metal foam sandwich structures against near-field blasts.

## 2. Experimental Procedures

### 2.1. Test Scheme and Test Specimens

**Figure 1:**
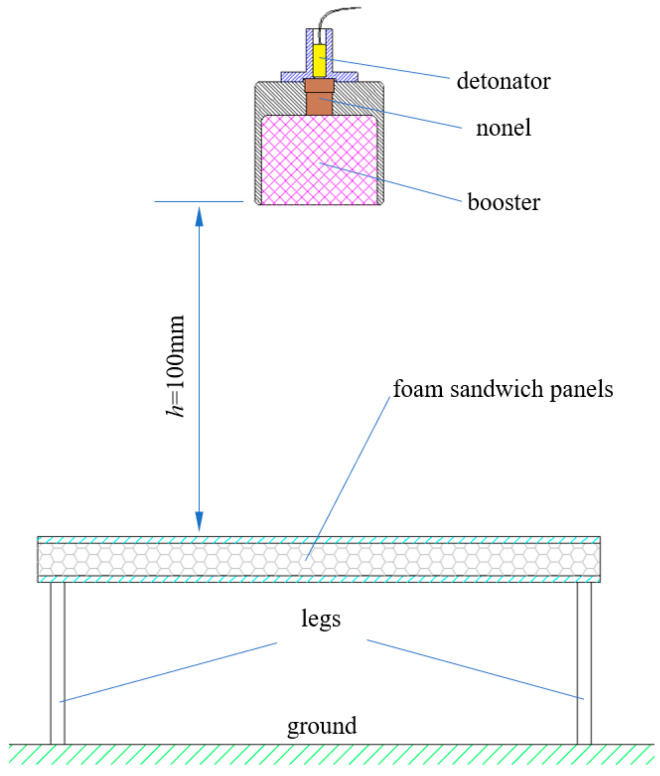
Schematic diagram of near-field blast test.

In order to study the dynamical response performance of the foam-nickel sandwich panels under the load of near-field blast, a near-field blast experimental platform was designed, as shown in Figure 1. Pins (legs) were used to fasten the sandwich panels by drilling holes in the edge circumference of the structure, and the entire structure was placed on level ground. The explosive suspended above the sandwich structure with the height of 100 mm was used to generate near-field blast load and was detonated by a bridge-wire type electric detonator that was connected to the explosive at one end and connected to the blasting device placed in the safety cover with a twisted pair of wires at the other end. The explosive used in the blast test is cylindrical-shaped 8701 explosive with a total charge weight of 35 g, and the explosive shell is covered with thin nylon material.

For the convenience of illustrating deformation characteristics of the sandwich panel under near-field explosion, strain gauges were pasted uniformly on the bottom panel of sandwich structure. After the blast test, the failure mode and deformation characteristics of the foam sandwich structure by the measurement of deformation–displacement and the analysis of strain data were obtained with the strain adopted device.

**Figure 2:**
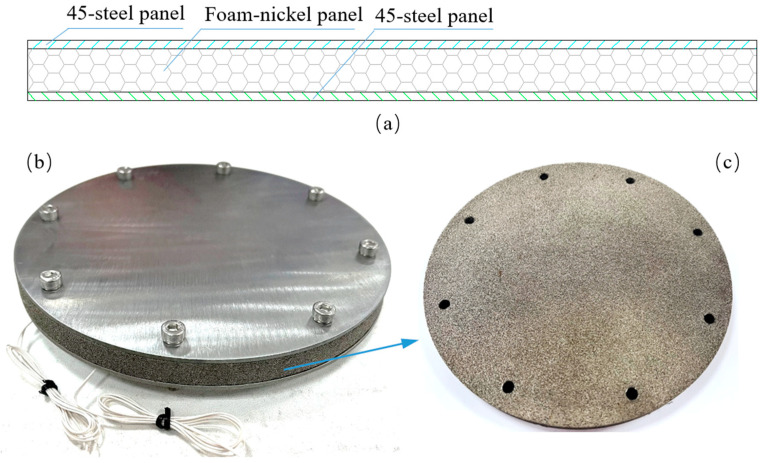
Foam-nickel sandwich panels: (**a**) schematic diagram of sandwich panels, (**b**) foam-nickel sandwich panels entity structure and (**c**) foam-nickel core material.

The sandwich structure based on foam-nickel core material has a diameter of 175 mm, which mainly consists of a top panel, foam-nickel core material and a bottom panel, as shown in Figure 2. In this structure, the top and bottom panel material both are made of 45 steels with the thickness of 2 mm, and the core material was made of open porosity foam-nickel prepared by foaming process with the total thickness of 10 mm, the density of 1.15 g/cm^3^, and the average spherical porosity of 0.3–0.5 mm. As shown in Figure 2c, the bubble in the foam-nickel material is spherical, and mounts of small holes are opened in the bulkhead to connect the spheres, which is conducive to sound absorption, filtration and sound attenuation. Three boards were placed in the order according to Figure 2 and tightened with 8 screws and nuts distributed circumferentially.

### 2.2. Test Setup

**Figure 3:**
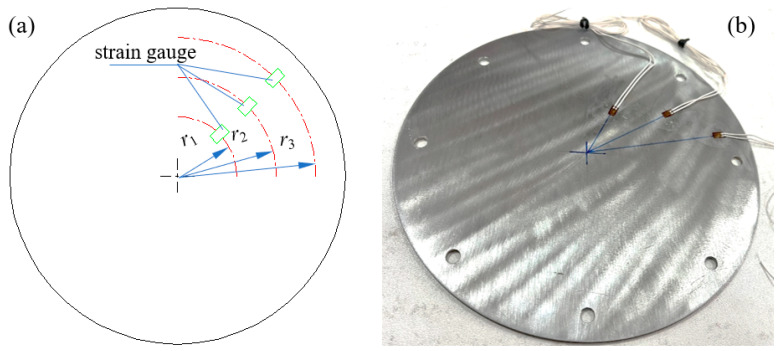
Strain test method under blast: (**a**) layout scheme of strain gauges, and (**b**) bonding method of strain gauges.

In order to test the strain of the bottom panel under near-field explosion, the BE120-3AA-P500 strain gauge with a resistance of 120 Ω was selected. Before the test, the three strain gauges located at radii of 30 mm, 50 mm and 70 mm, respectively, were pasted on the surface of the bottom panel by use of epoxy glue (CC-302A), as shown in Figure 3. The input end of the strain bridge box was connected to the strain gauge wire, and the output end of the bridge box was connected to the LZSDSP-8CH-5M strain adopted device.

**Figure 4:**
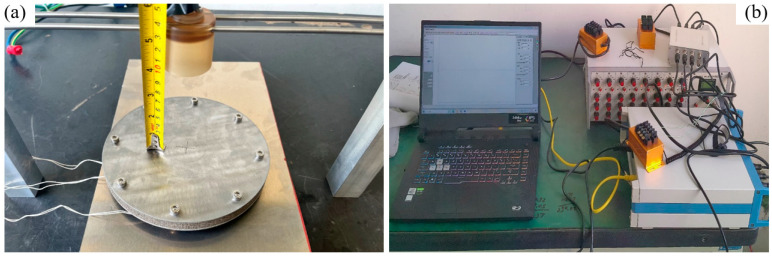
Blast test setup: (**a**) test platform, and (**b**) strain test system.

According to the above test scheme and methods, the test platform was built and the data adopted device was arranged, as shown in Figure 4.

### 2.3. Experimental Results and Discussion

#### 2.3.1. Damage Characteristics and Deformation Modes

**Figure 5:**
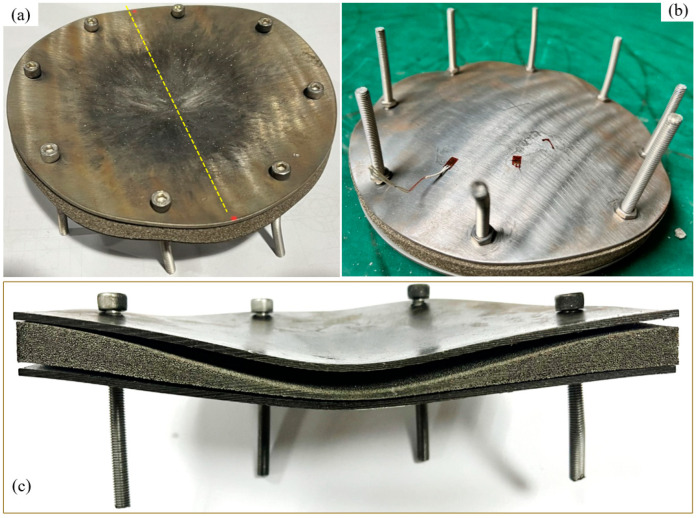
Views of foam-nickel sandwich panels after testing: (**a**) front view, (**b**) back view and (**c**) centre profile map along dashed line in (**a**).

After the test, the sandwich structure constructed on foam-nickel core material and steel plates was recovered and observed, as shown in Figure 5a,b. Under the action of explosion shock wave, the top panel of the sandwich protective structure was damaged and deformed, and the total structure underwent significant deformation, especially the foam-nickel panel, but no panel was broken through. Furthermore, the sandwich structure was dissected along the deformed bottom of the structure by wire-cutting method, and the damage and deformation details of the top panel and bottom panel were obtained, as shown in Figure 5c.

**Figure 6:**
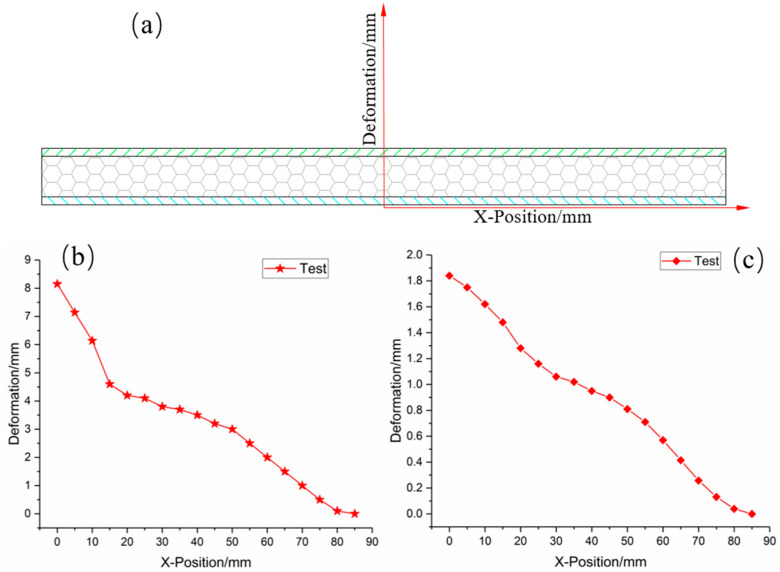
Deformation measurement: (**a**) schematic diagram of deformation measurement coordinates; (**b**) measurement results of foam-nickel core; (**c**) measurement results of bottom panel.

To illustrate quantitatively the deformation extent of the foam-nickel sandwich structure under the blast load, the reference coordinate system of the deformation measurement of sandwich structure is established, as shown in Figure 6a, where the zero point coordinate is selected as the centre of the circle of the bottom panel of the sandwich structure. When measuring, the deformation progression of the foam-nickel core material is measured with the centre as the zero point and 5 mm as the gradient. Figure 6b shows the position–deformation data of the foam-nickel core, displaying the rapid decline tendency of deformation amount with the increase in X location. Figure 6c shows the position–deformation data of the bottom panel as well.

The test results show that the sandwich structure based on foam-nickel core material can effectively protect and attenuate the explosive shock wave, which is consistent with previous experimental studies reported in Refs. [23,24,25]. During the explosion, the top panel is used to oppose the first order of explosive products, mainly shock waves, and to provide impact protection and structural support for the sandwich structure. In conclusion, the foam-nickel core is used to rapidly attenuate and absorb the explosion shock wave and provide a certain deformation space for the overall structure of the sandwich. Furthermore, the bottom panel again emits and attenuates the blast wave and provides structural support.

#### 2.3.2. Blast Resistance of Sandwich Panels

**Figure 7:**
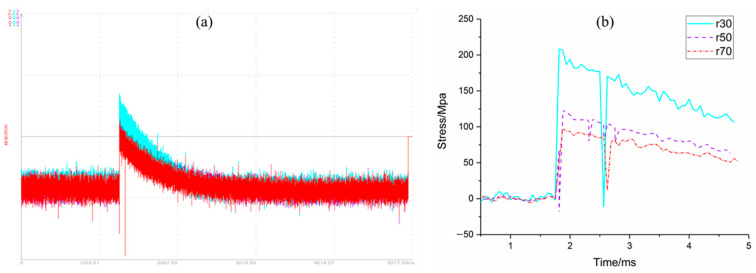
Stress versus time curve of bottom panel: (**a**) test curve, and (**b**) filtered curve.

The stress data transferred from test strain data collected by computer memorizer are shown in Figure 7. The strain data were adopted by strain gauges at the radii *r* = 30 mm, 50 mm and 70 mm of the bottom panel under the sandwich structure, respectively.

After explosion, under the action of shock wave, the strain gauge was detached from the bottom panel or the wire was cut, which leads to the real peak value possibly not being measured in the test data. The data in the second half of the curve should be interference signals coupled from the wire. Therefore, in the data analysis, the data of the rising edge of the signal are considered as valid data. The above signal was processed by progressive filtering (frequency of 25 kHz), as shown in Figure 7b.

## 3. Numerical Studies

### 3.1. Establishment of Finite Element Model

The liquid–solid coupled simulation model was established by virtue of the explicit dynamic software ANSYS/LS-DYNA R971, and the multi-substance Euler and Lagrangian method were applied on the calculation and analysis. This dynamic program is widely applied in high-speed impact and explosion problems.

**Figure 8:**
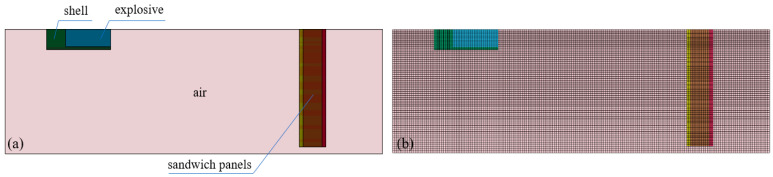
Model of shaped charge-blasting warhead: (**a**) geometric model, and (**b**) finite element model.

Similar to the experiments, a free-field explosion model of sandwich structure based on foam-nickel core material was established, which was composed of explosives, air, charge shell and sandwich protection structure. In the modelling of sandwich structure, the definition of contact algorithm between the steel plates and foam-nickel core material is set as Automatic Surface_to_Surface contact, which describes the free contact state for the actual sandwich structure. Moreover, the parameter values of Fs and Fd are set as 0.2 and 0.1 [27]. Fluid–structure coupling method was used for the calculation, where ALE algorithm was adopted on explosives and air, and the explosives were wrapped with air layer. Due to the bi-symmetry of the structural shape and blast load of the model, the free-field explosion model was established by using the 1/4 model, as shown in Figure 8. The geometric model was discretized into a finite element model of hexahedral elements, and the size of the sandwich protective structural element was controlled at about 1 mm.

**Figure 9:**
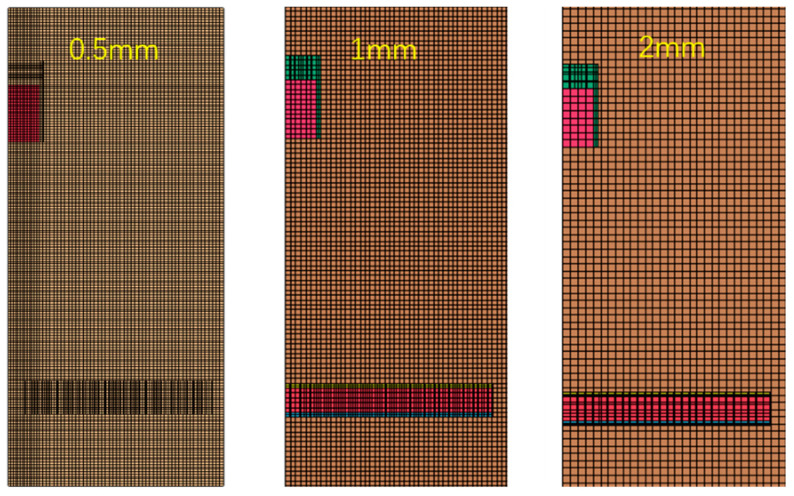
Different mesh sizes.

What is more, in order to ensure the mesh quality, we have created a mesh consistency analysis; that is, different numbers of finite element meshes are generated under different mesh sizes, and the finite element analysis is carried out. The mesh size and the corresponding number of meshes are shown in Table 1 and Figure 9 (1/4 model).

**Figure 10:**
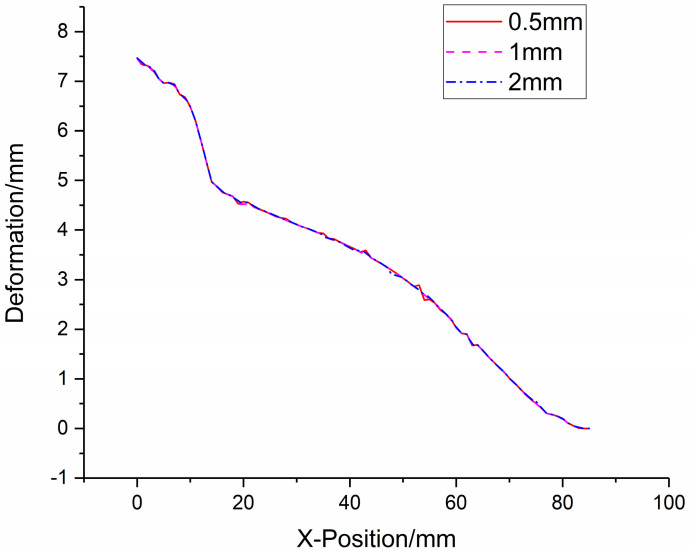
Deformation–position curve of foam-nickel core material of sandwich structure.

The deformation data of foam-nickel core material of sandwich structures under different mesh sizes are shown in Figure 9. It can be seen from deformation–position curves of the foam-nickel core material of sandwich structures under different mesh sizes that, when the mesh size is between 0.5 mm and 2 mm, the impact on the numerical simulation results is limited, as shown in Figure 10. In other words, on the premise of meeting the accuracy of numerical simulation, in order to reduce the amount of calculation as much as possible, it is reasonable to select the mesh size of 1 mm for numerical simulation in this paper.

### 3.2. Material Models

#### 3.2.1. Constitutive Equations of Explosive and 45 Steel

The shaped charge is 8701 explosive in the simulation calculation, which was simulated by using the MAT_HIGH_EXPLOSIVE_BURN model. The pressure and volume changes of the detonation products were described by JWL equation of state, as follows:

(1) $$p=A\left(1-\frac{\omega }{R_{1}V}\right)e^{-R_{1}V}+B\left(1-\frac{\omega }{R_{2}V}\right)e^{-R_{2}V}+\frac{\omega E}{V}$$

where *A*, *B*, *ω*, *R*_1_, *R*_2_ are the relevant coefficients demonstrating explosion, *E* is the unit volume energy, *V* is the initial relative volume. The material parameters of the explosive are listed in Table 2.

Air is simulated by using null matter material (*MAT_NULL), and the relationship between pressure and volume changes was described by LINEAR_POLYNOMIAL equation of state, as follows:

(2) $$p=C_{0}+C_{1}\mu +C_{2}\mu^{2}+C_{3}\mu^{3}+\left(C_{4}+C_{5}\mu +C_{6}\mu^{2}\right)E$$

The pressure of ideal gas can be expressed as

(3) $$p=\left(\gamma -1\right)\frac{\rho }{\rho_{0}}E$$

where *C*_4_ = *C*_5_ = *γ*−1, *ρ*_0_ is the initial density of air, and *ρ* is the current density of air. In this paper, *γ* = 1.4, and *ρ*_0_ = 0.0012 g/cm^3^.

The top and bottom panels of the sandwich protection structure are made of 45 steel with a thickness of 2 mm, using the JOHNSON_COOK model combined with the GRUNEISEN equation of state, which describes the behaviour of materials subjected to high strain rates, temperatures and pressure. The JOHNSON_COOK model can be expressed as follows:

(4) $$\sigma_{y}=\left[A+B{\left({\bar{\varepsilon }}^{p}\right)}^{n}\right][1+C\ln\left({\dot{\varepsilon }}^{*}\right)][1-{\left(\frac{T-T_{room}}{T_{m}-T_{room}}\right)}^{m}]$$

where *A*, *B*, *C*, *m* and *n* are material constants with *C* representing the strain rates effect, ${\bar{\varepsilon }}^{p}$ is the effective plastic strain, ${\dot{\varepsilon }}^{*}$ is the dimensionless strain rate, *T_m_* is the melting temperature of the material, *T* is the ambient temperature and *T_room_* is room temperature, respectively. The JC material parameters of 45 steel are listed in Table 3.

#### 3.2.2. Mechanical Performance of Foam-Nickel Material

The compressive mechanical properties tests of foam-nickel core material were carried out by a DDL-100 electronic universal testing machine, of which the maximum force value is 100 kN, and, regarding the force measurement accuracy, the error is less than 0.5%, deformation measurement accuracy error less than 0.5% and speed accuracy error less than 0.5%, respectively.

**Figure 11:**
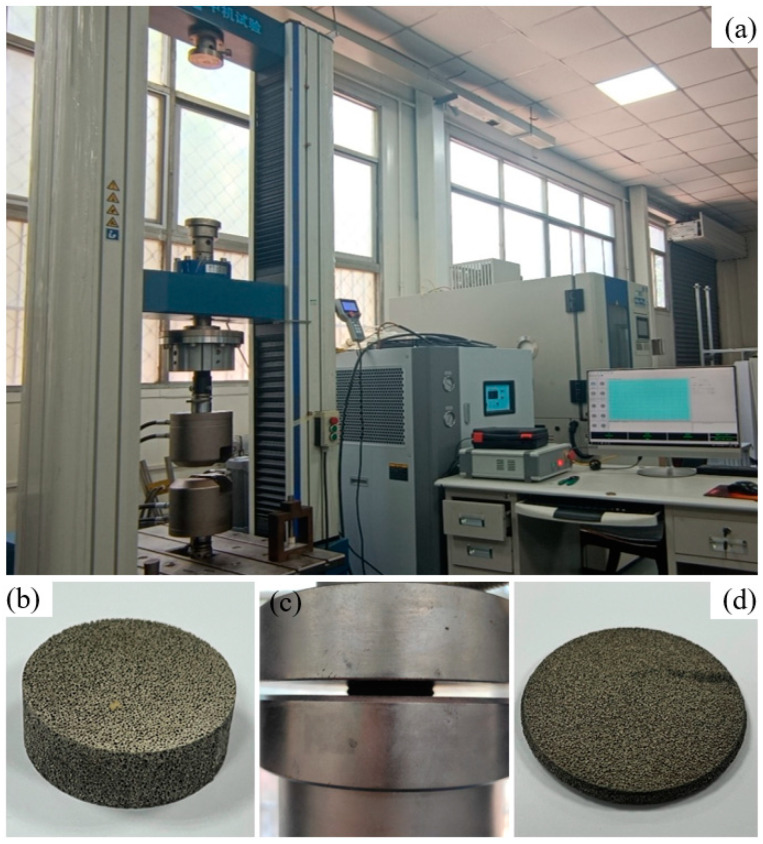
Compressive testing of foam-nickel: (**a**) DDL-100 electronic universal testing machine, (**b**) initial state of specimen, (**c**) compressive process and (**d**) deformation state of specimen after compressive test.

The size of cylindrical specimen is 30 × 10 mm, the density is 2.23 g/cm^3^ and the test process of mechanical properties of foam-nickel material is shown in Figure 11.

**Figure 12:**
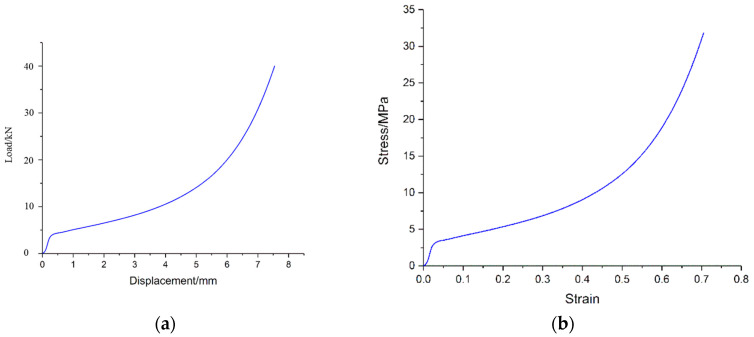
Quasi-static compressive performance of foam-nickel material: (**a**) load–displacement curve; (**b**) stress–strain curve.

As shown in Figure 12a, the load–displacement characteristics of foam-nickel material were obtained through the above quasi-static mechanical properties test with the loading rate of 10^−3^/s. It can be seen from the curve that, at the beginning, the foam-nickel material presents an ideal linear elastic deformation under the compression load, and the load increases uniformly with the displacement. Then, with the increase in displacement, the slope of the curve decreases obviously, and this process lasts for a long time, which is the main stage of energy absorption of foam-nickel. Finally, the foam-nickel structure was compacted, the structure density increases and the load increases rapidly with the increase in displacement, which is the densification stage of foam-nickel.

The mechanical properties of the porous foam material (named as S-1) can be illustrated in simulation analysis by using CRUSHABLE_FOAM constitutive model, which requires input stress–strain curve of material. Therefore, according to the load–displacement curve data obtained by laboratory test, the constitutive stress–strain curve can be illustrated, as shown in Figure 12b. Based on the curve data, several fundamental mechanical parameters can be obtained for the foam-nickel material: Young’s modulus is 120 MPa, Poisson’s ratio is 0.28 and yield strength is 5.35 MPa, as shown in Table 4. Furthermore, the strain rate effect of steel is represented by LCID (stress–strain curve), and, for the effect of volumetric strain in the foam material in the FE model, it is included in LCID, which defines yield stress as a function of volumetric strain.

### 3.3. Simulation Results and Discussion

#### 3.3.1. Structural Deformation Process and Stress Distributions

**Figure 13:**
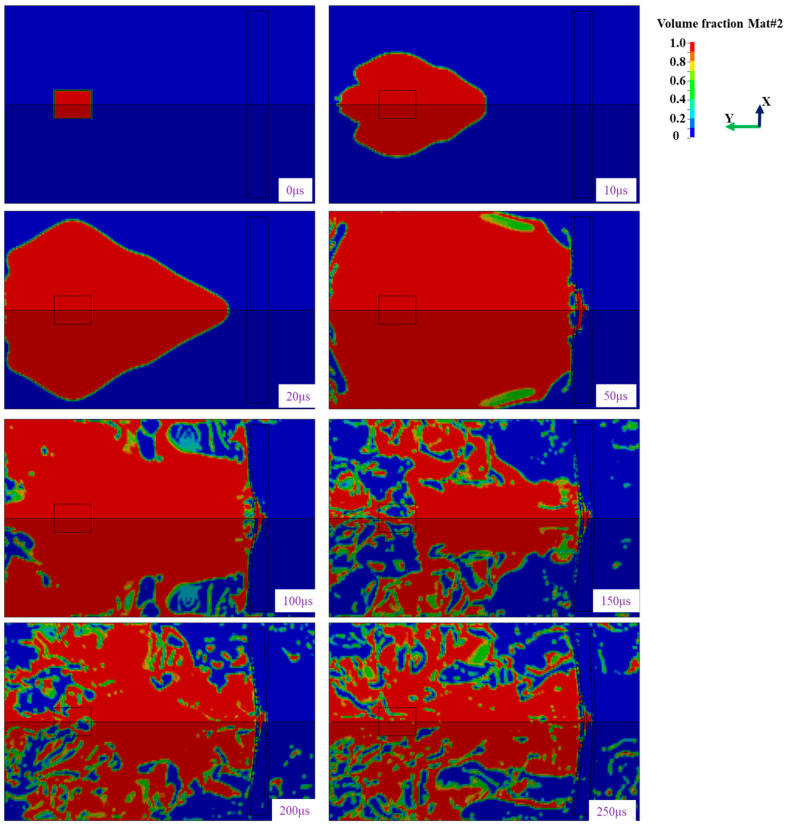
Effect diagram of explosive process.

According to the above established finite element model of sandwich structure based on foam-nickel core material, finite element software was used to calculate the response characteristics of sandwich structure in the process of shock wave transmission after explosive explosion. During calculation, the detonation moment is set to 0 μs, the result file is output once every 1 μs and the total calculation time is 500 μs. The effect diagram of the explosion simulation calculation process is shown in Figure 13.

**Figure 14:**
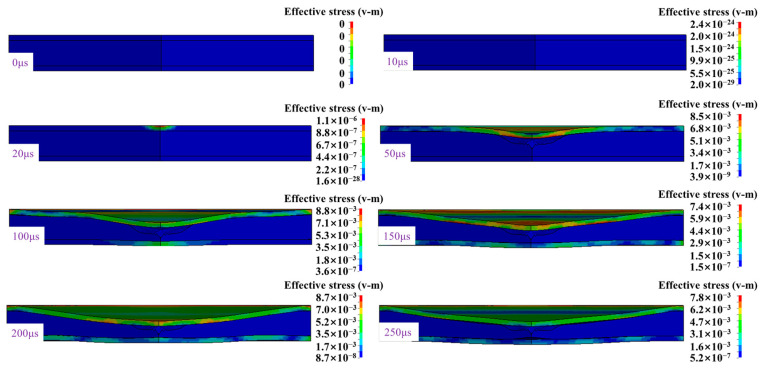
Deformation process of sandwich panels.

Under the action of blast load, the sandwich structure gradually deforms, and structural failure occurs in the bottom panel. The deformation diagram of sandwich protective structure is shown in Figure 14.

**Figure 15:**
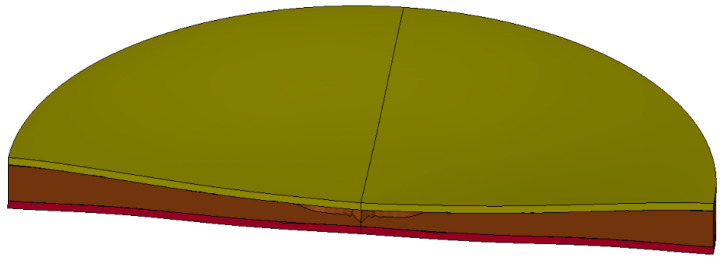
Deformation and failure diagram of sandwich protective structure.

After the calculation, the deformation state of the sandwich structure is shown in Figure 15. The sandwich structure as a whole has obvious deformation, the top panel has plastic deformation and the local compression deformation of the foam-nickel core material reaches 90%.

**Figure 16:**
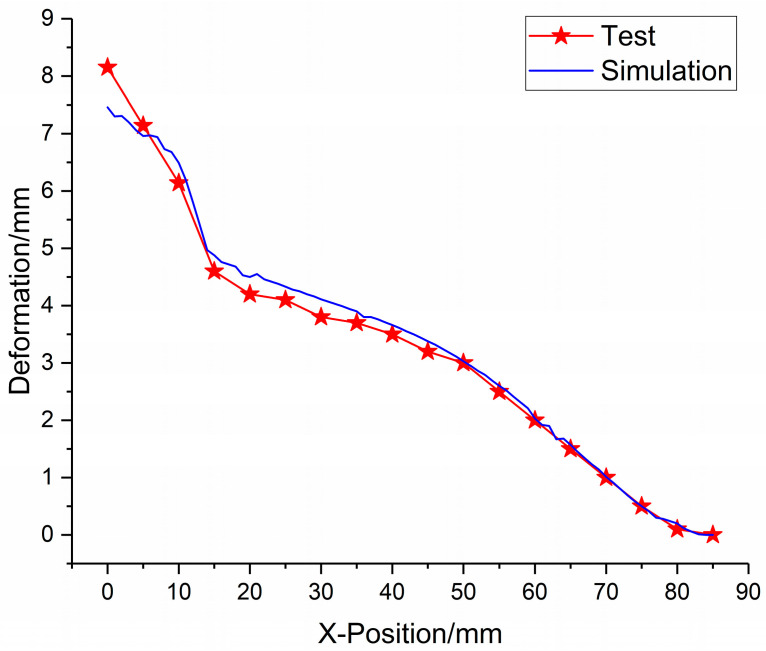
Comparison between simulation and test data of foam-nickel core material position–deformation.

The deformation data of foam-nickel core material of sandwich structure were measured, and the deformation–position curve of sandwich structure under blast load was obtained. The comparison and analysis of the simulation data and the test data of the position–deformation of foam-nickel core material are shown in Figure 16.

**Figure 17:**
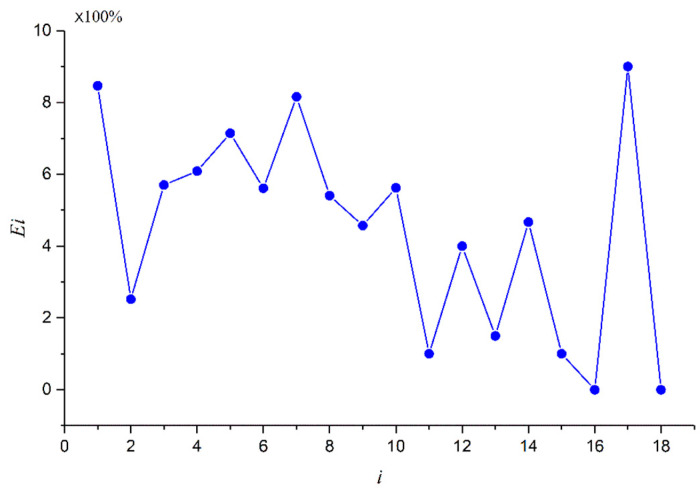
Position–deformation simulation error of each measuring point.

Calculating the error *E_i_* of deformation–position at each measurement point:

(5) $$E_{i}=\frac{\left|D_{i}^{t}-D_{i}^{s}\right|}{D_{i}^{t}}$$

where $D_{i}^{t}$ is the tested data at the *i*th point, $D_{i}^{s}$ is the simulated data at the *i*th point and the relative results are shown Figure 17. Meanwhile, the comprehensive error *E* of deformation for foam-nickel core material can be expressed as

(6) $$E=\frac{\sum_{1}^{n}E_{i}}{n}$$

According to the error *E_i_* of the deformation–position simulation for each measuring point, it can be obtained from Formula (6) that the comprehensive error is *E* = 4.47%.

**Figure 18:**
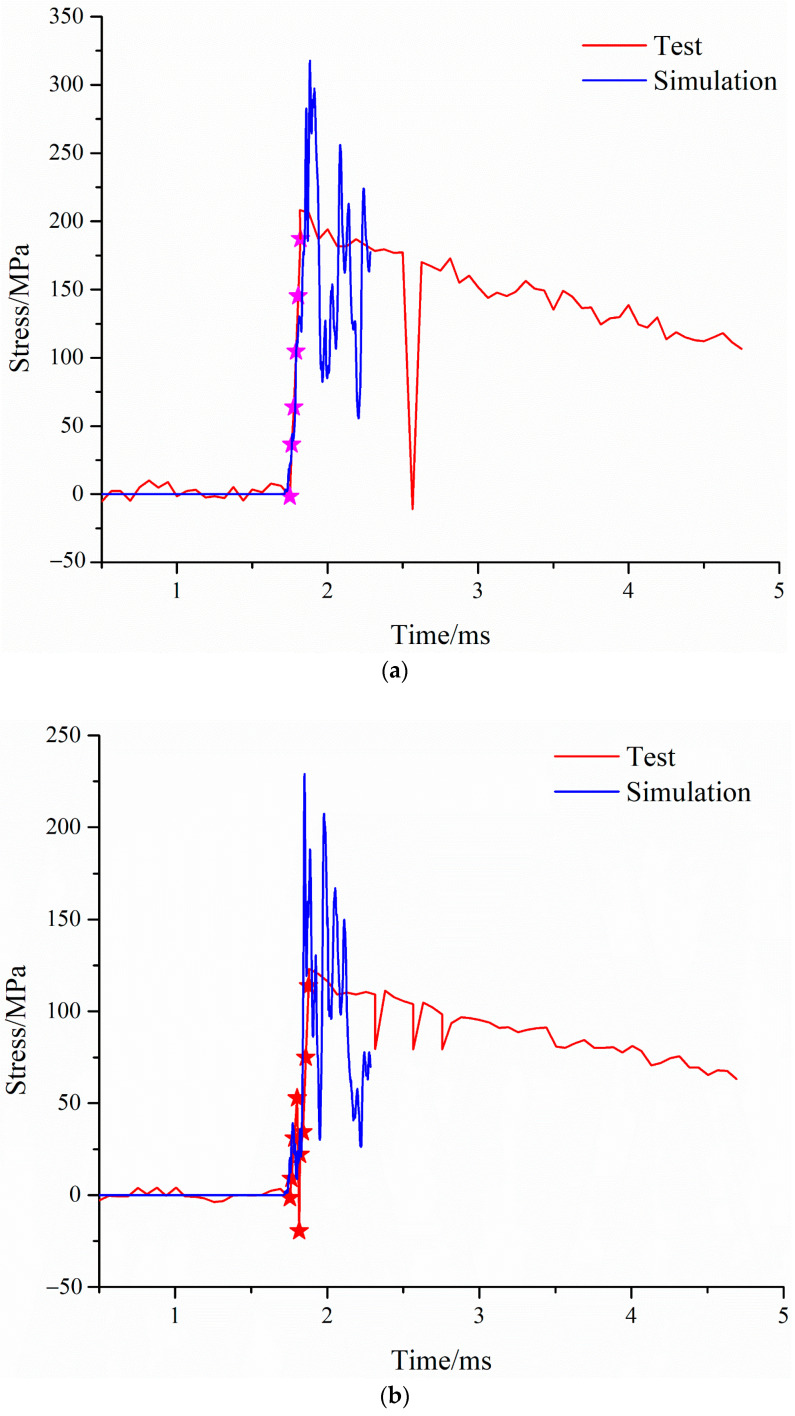

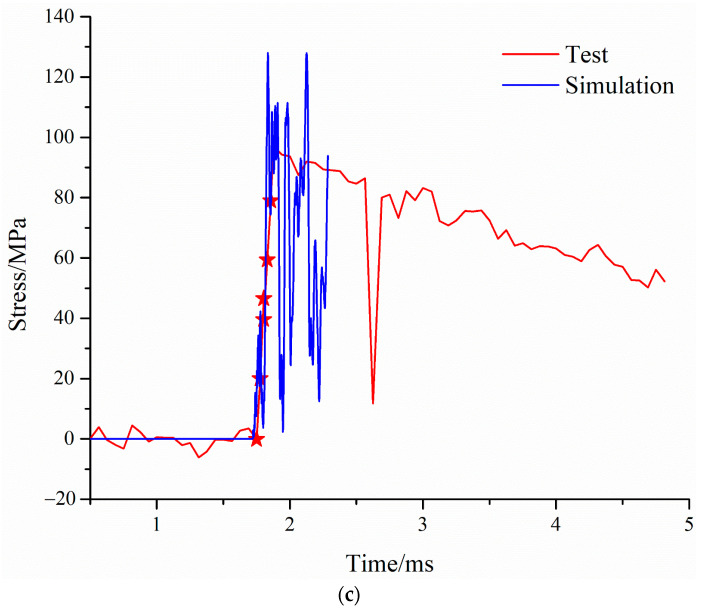
Stress distribution characteristics of bottom panel compared with simulation and test. (**a**) *r* = 30 mm. (**b**) r = 50 mm. (**c**) r = 70 mm.

After the end of the simulation calculation, the stress–time characteristic curve of the bottom panel of the sandwich structure under the blast load was established, and the simulation data of the stress–time characteristics of the bottom panel were compared with the test results, as shown in Figure 18. As mentioned above, after explosion, under the action of shock wave, the strain gauge was detached from the bottom panel or the wire was cut. Therefore, the real peak value may not be captured in the measured data, and the data in the second half of the curve should be interference signals coupled in from the wire. Furthermore, in the progressive data analysis, it is considered that the part of the rising edge of the signal is valid data. Therefore, when comparing and analysing the simulation data of the stress–time characteristics of the bottom panel with the test results, the rising edge part of the signal, that is, the data during the period of 1.75 ms to 1.82 ms (the part marked ★ in Figure 18) were only considered in this paper.

As can be seen from Figure 18, the stress–time characteristics simulation data of the bottom panel are consistent with the rising edge trend of the test curve, indicating that the simulation data are highly consistent with the test results.

In summary, the analysis results show that the sandwich structure model based on foam-nickel core material under the action of blast load is reasonable and accurate, and the established model has a high degree of accuracy, which can be used for the optimal design of sandwich structure based on foam-nickel core material.

#### 3.3.2. Comparisons of Blast Protective Performance for Foam-Nickel Panels with Different Porosities

**Figure 19:**
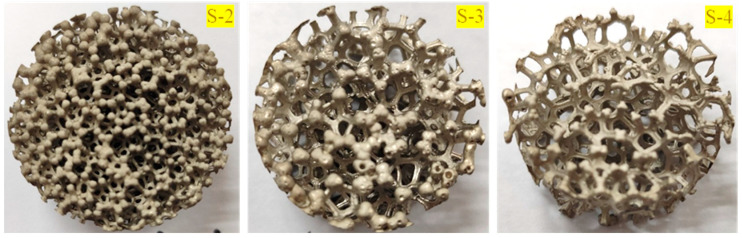
Compression test specimens of foam-nickel material with three different porosities (*n* = 85% for S-2, *n* = 90% for S-3 and *n* = 95% for S-4).

The above experimental and simulation results show that the sandwich structure remains intact without generating fragments, collapse or breakthrough at any positions of the structure, which can be regarded as no failure in existence for the entire sandwich structure. For the purpose of investigating the protective performance of the sandwich structure based on foam-nickel material, the other three kinds of specimens of foam-nickel material were prepared with the density of 2.23 g/cm^3^ and a porosity of 75%, the density of 1.30 g/cm^3^ and the porosity of 85% and the density of 0.90 g/cm^3^ and the porosity of 90%, respectively. The mechanical properties of the test specimens created from the above-mentioned foam-nickel material are shown in Figure 19.

**Figure 20:**
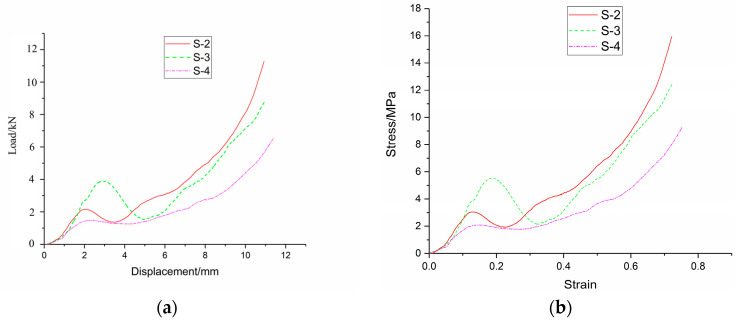
Compression test results of foam-nickel materials with three different porosities: (**a**) load-displacement curve; (**b**) stress–strain curve.

The quasi-static compression testing platform with a DCL-100 electronic universal testing machine was applied to test the load–deformation data of three types of foam-nickel specimens, respectively, as shown in Figure 20a. Stress–strain curves of three types of constitutive models of foam-nickel were established according to the load–deformation test data, as shown in Figure 20b.

**Figure 21:**
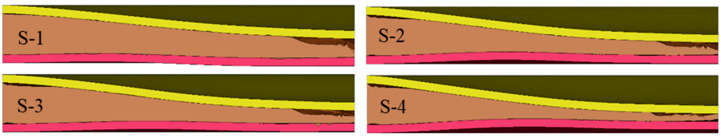
Deformation diagram of 4 types of foam-nickel cores under blast load, and ‘S-*n*’ indicates four different porosities in which the corresponding porosities are 75%, 85%, 90% and 95% for S-1, S-2, S-3 and S-4 respectively.

According to the above established certain types of finite element models of sandwich structure based on foam-nickel core material, except for replacing the constitutive model parameters of foam-nickel core material, the remaining parameters remain unchanged, and the deformation of foam-nickel core material of four types of sandwich structures under blast load was calculated, as shown in Figure 21.

**Figure 22:**
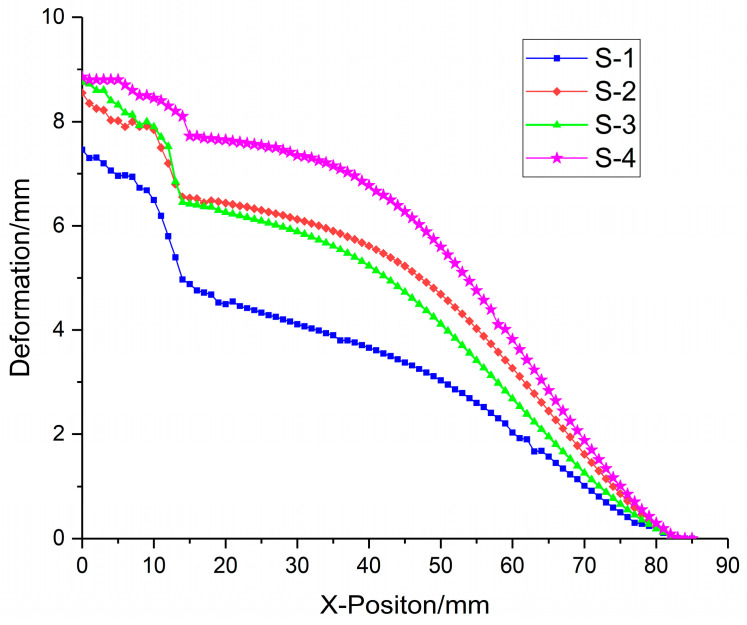
Deformation data diagram of foam-nickel core for 4 kinds of sandwich structures.

Under the blast load, the foam-nickel core protected by the sandwich structure absorbs the blast load energy through its own deformation. The deformation–position curve of the foam-nickel core of the sandwich structure is shown in Figure 22. When the density of foam-nickel core is 2.23 g/cm^3^ and the porosity is 75%, the deformation range of foam-nickel core is 0.011–7.461 mm. When the density of foam-nickel core is 1.30 g/cm^3^ and the porosity is 85%, the deformation range of foam-nickel core is 0.015–8.653 mm. When the density of foam-nickel core is 0.90 g/cm^3^ and the porosity is 90%, the deformation range of foam-nickel core is 0.012–8.692 mm. When the density of foam-nickel core material is 0.45 g/cm^3^ and the porosity is 95%, the deformation range of foam-nickel core material is 0.018–8.850 mm. Moreover, with the decrease in density and the increase in porosity, the deformation of foam-nickel core material increases gradually, and the relative comparison results of foam-nickel materials with four porosities are listed in Table 5.

**Figure 23:**
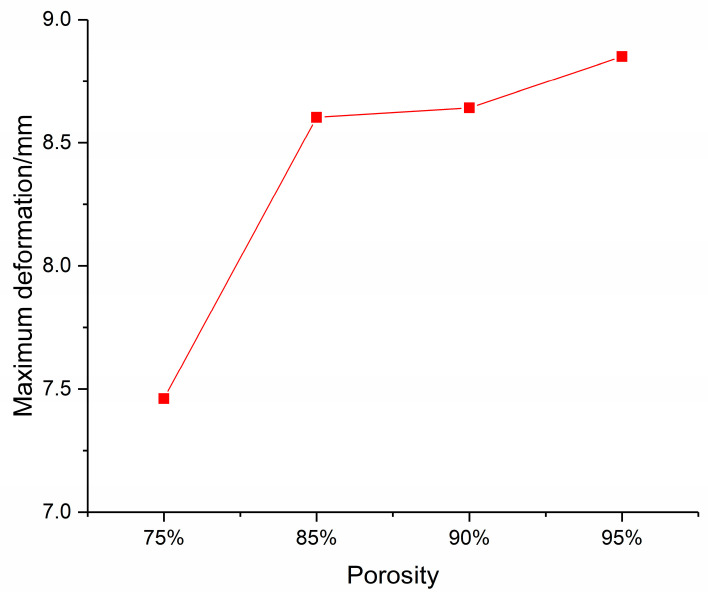
Relationship between core deformation characteristics and porosity.

As shown in Figure 23, the comparison of deformation characteristics of four kinds of core materials shows that the maximum deformation of foam-nickel core material gradually increases with the gradual decrease in the density and the gradual increase in the porosity. The deformation of foam-nickel core with 85% porosity is basically the same as that of 90% porosity.

**Figure 24:**
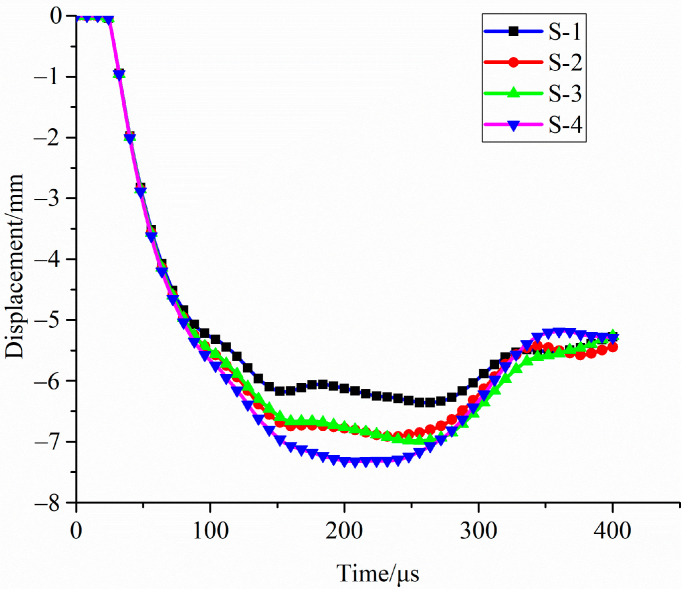
Deformation state of top panel for 4 kinds of sandwich structures.

After the simulation, the deformation–time characteristic curve of the upper panel of sandwich structure under blast load was established. Under blast load, the deformation time of the top panel with the protection of sandwich structure based on foam-nickel core material is shown in Figure 24.

As shown in Table 6, the maximum deformation of the top panel is 6.355 mm and the maximum deformation moment occurs at 264 μs for the foam-nickel core material with the density of 2.23 g/cm^3^ and the porosity of 70%; the maximum deformation of the upper panel is 6.920 mm and the maximum deformation moment occurs at 236 μs for the foam-nickel core material with the density of 1.30 g/cm^3^ and the porosity of 85%; the maximum deformation of the upper panel is 6.977 mm and the maximum deformation moment occurs at 261 μs for the foam-nickel core material with the density of 0.90 g/cm^3^ and the porosity of 90% and the maximum deformation of the top panel is 7.323 mm and the maximum deformation moment occurs at 207 μs for the foam-nickel core material with the density of 0.45 g/cm^3^ and the porosity of 95%.

**Figure 25:**
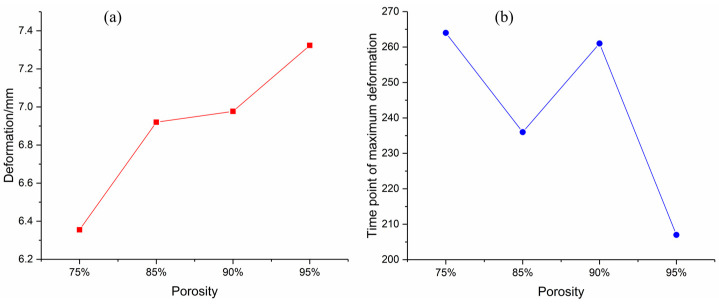
Relationship between deformation of top panel and porosity of foam-nickel core: (**a**) deformation porosity characteristics; (**b**) time point of maximum deformation porosity characteristics.

As shown in Figure 25, the comparison of the deformation characteristics of the top panel for the four core materials shows that the maximum deformation of the top panel gradually increases with the gradual decrease in the density and the gradual increase in the porosity for the foam-nickel core material. The deformation of the top panel of the foam-nickel core material with 85% porosity and 90% porosity is basically the same. There are fluctuations when the maximum deformation occurs.

In fact, the sandwich structure subjected to near-field blast load can be simplified as the impact bending problem of simply supported plates. Therefore, not only can the deformation quantity of the structure be characterized by using the displacement at the centre point of blast generation but can also be characterized by using the rotation angle of the end of the structure. The expected support rotation angle *θ* typically results from a combination of the maximum displacements *y*_max_ and of the panel diameter *D*, that is [29,30,31]:

(7) $$\theta =\tan^{-1}\left(\frac{y_{max}}{0.5D}\right)$$

The rotation angle values of the top panel and the bottom panel shall be calculated by using above formula, and the relevant results are also listed in Table 7.

On the other hand, the calculated rotation angle values were compared with a set of limit values provided by standards, for example, the UFC-3-340-02 provisions [32,33]. In these references, the support rotation criterion is recommended as a suitable design method for blast-loaded steel members (see Table 8). According to the allowable rotation values of Table 7, it is clear that the rotation angles of the top panel of four kinds of core materials are all greater than 4°. Therefore, the deformation characteristics of the top panel of 4 kinds of core materials all fall into high damage level. Meanwhile, the bottom panel of 4 kinds of core materials are all between 1° and 2°, which just means the deformation characteristics of the bottom panel all fall into medium damage level.

**Figure 26:**
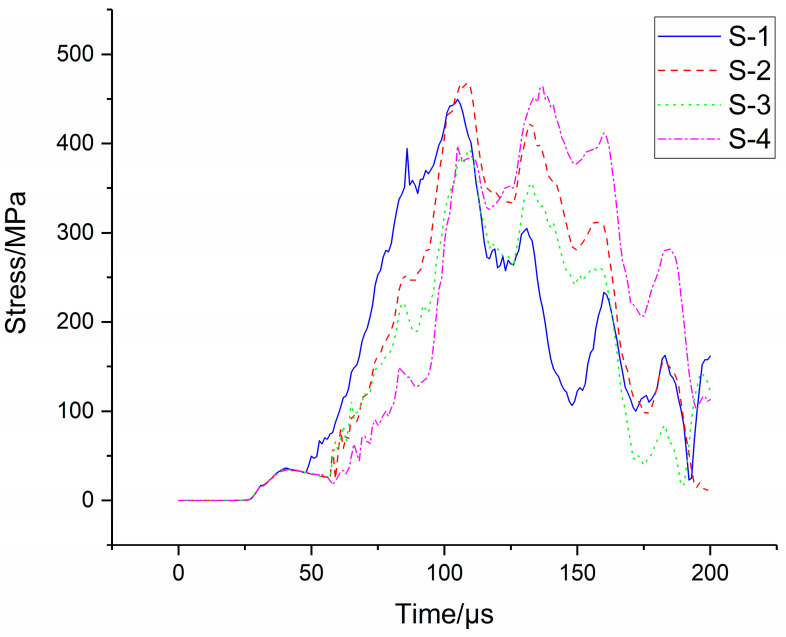
Stress distribution of bottom panel.

Under blast load, the work stress of the bottom panel protected by sandwich structure based on foam-nickel core material is shown in Figure 26.

As shown in Table 9, the peak stress of the bottom panel is 449.49 MPa, correlating the peak moment at 105 μs and the pulse width of 140 μs for the foam-nickel core material with the density of 2.23 g/cm^3^ and the porosity of 75%; the peak stress of the bottom panel is 467.63 MPa, correlating the peak moment at 109 μs and the pulse width of 132 μs for the foam-nickel core material with the density of 1.30 g/cm3 and the porosity of 85%; the peak stress of the bottom panel is 391.32 MPa, correlating the peak moment at 110 μs and the pulse width of 117 μs for the foam-nickel core material with the density of 0.90 g/cm^3^ and the porosity of 90% and the peak stress of the bottom panel is 464.43 MPa, correlating the peak moment at 137 μs and the pulse width of 177 μs for the foam-nickel core material with the density of 0.45 g/cm^3^ and the porosity of 95%.

**Figure 27:**
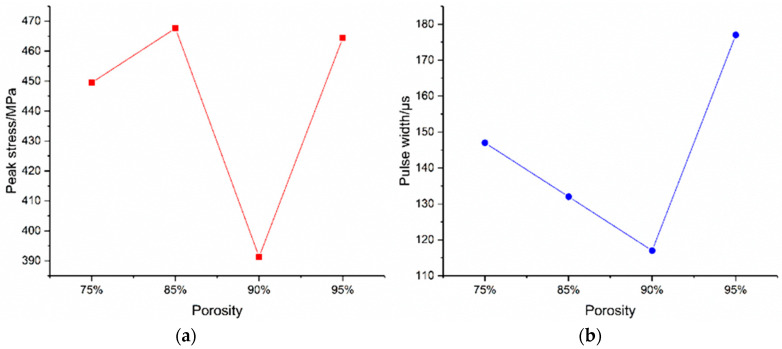
Relationship between stress of bottom panel and porosity of foam-nickel core. (**a**) Relationship between peak stress and porosity. (**b**) Relationship between stress pulse width and porosity.

As shown in Figure 27, the comparison of the work stress characteristics of the bottom panel of the four core materials shows that the peak stress of the bottom panel first increases and then decreases as the density and porosity of the foam-nickel core material gradually decrease. The pulse time decreases first and then increases. The work stress of the panel reaches the minimum value for the foam-nickel core material density of 0.90 g/cm^3^ and the porosity of 90%, which is the best choice of core material for the sandwich protection structure.

## 4. Conclusions

In this paper, the response of the foam-nickel sandwich panels under near-field blast loading was systematically investigated by experimental tests and numerical simulation. The deformation and stress of the typical structure of foam-nickel sandwiches with different porosity under near-field blast load were analysed in detail. The influence of several key parameters, such as near-field explosion pulse, panel material configuration and foam porosity, on the anti-explosion performance of sandwich panels was discussed. Several beneficial conclusions are as follows. On the other hand, there are some significant issues that could be investigated in the future, including near-field explosion protection effect or response rule of sandwich structure based on foam-nickel core under the various explosive charge ranges.

(1) The data of the simulation curve of the position–deformation characteristics of the foam-nickel core are similar to the measured curve; the comprehensive error is 4.47%, and the characteristics of the stress–time characteristics of the simulation data are similar to the measured data. The results obtained by the experiment and the simulation are in good agreement, indicating that the numerical model established in this paper is reasonable.

(2) The test and simulation results show that the sandwich structure based on foam-nickel core material can effectively protect and attenuate the blast load. The top panel can be used to protect the first level of explosive products, such as shock waves, fragments, etc., to provide protection and structural support for the sandwich structure; the foam-nickel core material is used to rapidly attenuate and absorb the explosion shock wave and provide a certain deformation space for the overall sandwich structure; the lower panel re-emits and attenuates the blast wave and provides structural support.

(3) As the density of foam-nickel core decreases and the porosity increases, the maximum deformation of foam-nickel core increases gradually. The deformation of foam-nickel core with 85% porosity is basically the same as that of 90% porosity.

(4) With the decrease in the density and the increase in porosity of foam-nickel core material, the maximum displacement and deformation of the top panel increase gradually. The deformation of the top panel of the foam-nickel core material with 85% porosity and 90% porosity is basically the same, and there are fluctuations when the maximum deformation occurs.

(5) With the decrease in the density and the increase in porosity of foam-nickel core material, the peak stress of the bottom panel first increases and then decreases, and the pulse time decreases first and then increases. When the density of foam-nickel core material is 0.90 g/cm^3^ and the porosity is 90%, the working stress of the panel is minimum, which is the best choice of core material for a sandwich protection structure.

## Figures and Tables

**Table 1:** Mesh size and corresponding mesh quantity.

**Mesh Size/mm**	0.5	1	2
**Number of grids**	1,026,479	796,974	312,378

**Table 2:** Material parameters of 8701 explosive [28].

ρ (kg/m^3^)	*D* (m/s)	*A* (GPa)	*B* (GPa)	*ω*	*R*_1_	*R*_2_	*E* (GPa)	*V*
1680	8800	852.4	18.02	0.38	4.5	1.3	8.5	1

**Table 3:** Material parameters of 45 steel.

*ρ*_0_ (kg/m^3^)	*E*/GPa	*A*/MPa	*B*/MPa	*n*	*C*	*m*
7800	47.7	507	320	0.28	0.064	1.06
*C_p_* (J/kg∙K)	*T_m_* (K)	*D*_1_	*D*_2_	*D*_3_	*D*_4_	*D*_5_
469	1795	0.1	0.76	1.57	0.005	−0.84

**Table 4:** Material parameters of foam-nickel material.

*ρ*_0_ (g/cm^3^)	*E*/MPa	*μ*
2.23	120	0.28

**Table 5:** Comparison of deformation characteristics of foam-nickel core materials.

Specimen	Density (g/cm^3^)	Porosity	Maximum Deformation/mm
S-1	2.23	75%	7.461
S-2	1.30	85%	8.653
S-3	0.90	90%	8.692
S-4	0.45	95%	8.850

**Table 6:** Comparison of deformation characteristics of the top panel of 4 kinds of core materials.

Specimen	Density (g/cm^3^)	Porosity	Maximum Deformation/mm	Corresponding Moment/μs
S-1	2.23	75%	6.355	264
S-2	1.30	85%	6.920	236
S-3	0.90	90%	6.977	261
S-4	0.45	95%	7.323	207

**Table 7:** Calculations of rotation angle on both the top and bottom panel.

Specimen		Maximum Deformation/mm	Rotation Angle/°
S-1	Top panel	6.355	4.276
	Bottom panel	1.756	1.183
S-2	Top panel	6.920	4.654
	Bottom panel	1.852	1.248
S-3	Top panel	6.977	4.692
	Bottom panel	1.870	1.260
S-4	Top panel	7.323	4.924
	Bottom panel	1.982	1.336

**Table 8:** Expected damage level for steel frame members according to UFC provisions [33] based on support rotation.

	Component	Level of Damage		
		Low	Medium	High
Maximum allowable support rotation (in degrees)	Steel frame members (without significant compression) ^a^	1°	2°	4°

^a^ “Significant compression” is associated with an axial compressive load that exceeds up to 20% of the dynamic axial capacity of the member.

**Table 9:** Comparison of stress distribution of bottom panel for 4 kinds of foam-nickel core materials.

Specimen	Density (g/cm^3^)	Porosity	Peak Stress/MPa	Corresponding Moment/μs	Stress Pulse Width/μs
S-1	2.23	75%	449.49	105	140
S-2	1.30	85%	467.63	109	132
S-3	0.90	90%	391.32	110	117
S-4	0.45	95%	464.43	137	177

## Data Availability

No new data were created or analyzed in this study. Data sharing is not applicable to this article.
